# When magic happens. An interesting study of symptoms

**DOI:** 10.1192/j.eurpsy.2023.1629

**Published:** 2023-07-19

**Authors:** P. García Vázquez, C. Vilella Martin, E. Seijo Zazo, E. Gil Martín, C. Alvarez Vazquez

**Affiliations:** 1 Psychiatry, HUCA, Oviedo; 2 CAULE, Leon; 3 HUCA, Oviedo, Spain

## Abstract

**Introduction:**

Among the cases usually referred to Pain Clinics, the existence of cases of medically unexplained symptoms (MUS) is frequent, which are defined as those physical symptoms that present little or no basis that they respond to an underlying organic disease or that even when organic disease exists the symptoms are inconsistent or disproportionate to it.

**Objectives:**

The main objective of this study is to know the proportion of patients who present MUS among those referred to a Pain Clinic. Secondarily, an attempt will be made to classify those patients with MUS in different diagnostic categories.

**Methods:**

Observational study. All those patients referred to the Pain Unit of the Complejo Asistencial Universitario de León for 18 months were included in the study. All patients are evaluated in real clinical conditions, without any experimental control of variables, initially by a multidisciplinary team made up of Anesthesiologist, Psychiatrist and Rehabilitator and, in those who suspect a MUS condition, individually by part of psychiatry in order to confirm and characterize the syndrome.

**Results:**

462 patients were evaluated in a multidisciplinary way. 174 (37.7%) were male and 288 (62.3%) females. The mean age was 59.06 + 16.30 years. After the multidisciplinary assessment, two groups of patients were formed, one of 313 patients (67.75%) in whom there was no suspicion of MUS and the other of 149 patients (32.25%) in whom the existence of a MUS condition was suspected and who were referred for evaluation by psychiatric interview. After the psychiatric interview, it is observed that psychopathological and social factors explain the painful condition in 23.7% of the cases. The diagnoses found were Somatoform Disorders and Central Sensitization (N = 49, 10.6%), Malingering (N = 23, 5%), Factitious Disorders (N =21, 4.5%) and Other Diagnoses (N = 38, 8.2%).

**Conclusions:**

The psychological and social factors are relevant to explain the condition of up to

23.7% of the patients referred to the pain unit.

**Disclosure of Interest:**

None Declared

